# Validation of a Passive Solar Drying System Using Pineapple

**DOI:** 10.3390/foods13193081

**Published:** 2024-09-27

**Authors:** Katie Kuhn, Charles Strnad, Paige Bowman, Keoni Young, Emma Kroll, Anna DeBruine, Ian Knudson, Michael Navin, Qingsu Cheng, Michael Swedish, Wujie Zhang

**Affiliations:** 1 Chemical and Biomolecular Engineering Program, Physics and Chemistry Department, Milwaukee School of Engineering, Milwaukee, WI 53202, USA; kuhnk@msoe.edu (K.K.); strnadc@msoe.edu (C.S.); bowmanp@msoe.edu (P.B.); keoyoun109@gmail.com (K.Y.); emekroll@gmail.com (E.K.); anndebrul@gmail.com (A.D.); ianjknudson@gmail.com (I.K.); 2 Physics and Chemistry Department, Milwaukee School of Engineering, Milwaukee, WI 53202, USA; navin@msoe.edu; 3 Department of Biomedical Engineering, University of Wisconsin-Milwaukee, Milwaukee, WI 53211, USA; chengq@uwm.edu; 4 Mechanical Engineering Department, Milwaukee School of Engineering, Milwaukee, WI 53202, USA; swedish@msoe.edu

**Keywords:** drying, freeze-drying, pineapple, preservation, solar drying

## Abstract

Wasted produce is a pertinent issue in agriculture, with billions of tons of produce going to waste even before it hits markets. Specifically, in Sub-Saharan Africa (SSA), nearly half of all produce is lost before market. To combat this, the Agricycle^®^ passive solar drier was designed to provide a cost-effective method of drying fruit for preservation. Using a psychrometric chamber to simulate the SSA environment, vitamin C, total phenolic contents, and iron tests were conducted, along with microbial content determination, water content determination, dissolved solids testing, and color and microstructure analyses to validate passive solar drying, comparing the results to freeze-dried samples. Nutritional contents were comparable between fresh, freeze-dried, and solar-dried samples, with a loss in vitamin C (statistically significant), total phenolic contents, and dissolved solids during solar drying. The microbial analysis for solar-dried samples was below standard limits, and the water content in the solar-dried samples was ~10% w.b. (<20% w.b.) compared to ~3% w.b. of the freeze-dried samples. Although having comparable vitamin C, total phenolic contents, and iron values, freeze-dried and solar dried samples showed very different colors and microstructures based on colorimetry and SEM imaging. In conclusion, the Agricycle^®^ passive solar drier is a promising alternative approach for food preservation.

## 1. Introduction

One major societal issue that affects people worldwide is food waste. Globally, at least 1 billion tons of food is wasted annually [1], and in 2022, the number of food-insecure individuals increased to 1.3 billion [2]. Food can be discarded for a variety of reasons; it can be thrown out due appearance, defects, damaged packaging, or from soiling. Efficient food preservation is crucial for maximizing daily production. Methods such as freeze-drying or oven drying are excellent for food preservation due to their efficiency in removing water from foods. Currently, both freeze-dried intact fruits and restructured fruits are in the food market [3]. However, they can be expensive, as even a home-sized freeze-dryer can cost thousands of dollars. Alongside that, the regions of the world that suffer the most from food insecurity often have underdeveloped or nonexistent electrical systems [4], which significantly limits their options for food preservation processes. Solar drying is a commonly used as an effective and cheap method for preserving produce, utilizing the energy of sunlight.

Solar drying, while it may be cost-effective, bears several complications. First, drying solely using sunlight, depending on ambient temperature and humidity, may require multiple days to fully dry fruit to the necessary standards. The prolonged time may lead to unwanted microbial growth and opportunities for pests to access and consume the produce. The use of direct sunlight exposure to dry foods also harms volatile compounds within the produce, which can lead to the loss of nutritional contents such as vitamin C, and the loss of color due to photobleaching.

Passive solar drying utilizes convective heat transfer (through air circulation) between hot air and the food material to achieve drying [5]. Instead of sunlight directly contacting the produce, black, food-grade polyvinyl acrylamide is used to absorb sunlight and heat into the trays. Perforated holes and corrugation within the top and bottom trays allow hot air to rise and circulate through the system, removing moist air and allowing fresh air to cycle through. The holes are large enough to allow substantial air flow, but small enough to deter pests such as mice from consuming the fruit left out to dry.

A pilot validation of the Agricycle^®^ passive solar drier was performed using apples and showed great promise [5]. However, the pilot study only controlled the temperature, not the humidity. The comparison between passive solar drying and other drying methods, such as freeze-drying, was not conducted. The goal of this project was to further validate the Agricycle^®^ passive solar drying system—to control both temperature and humidity and perform more intensive analyses. Pineapple was chosen for this validation project because it is a common crop in Sub-Saharan Africa. It grows readily and is economically vital to many small farmers in the region. Pineapple is favored by consumers for its sweet flavor and crisp texture in addition to its health benefits such as anti-inflammatory properties [6,7]. However, pineapple is quite vulnerable to browning, decomposition, and spoilage from microbes such as yeasts, molds, and bacteria due to its high water content; therefore, a need is present for an effective preservation method [6,7,8]. Additionally, pineapples are easily accessible and can be sliced consistently for structural analysis. In this study, using pineapples, the effectiveness of passive solar drying compared to freeze-drying was investigated by determining water content, nutritional contents, structure, and visual appeal. Freeze-dried pineapple samples were used as a benchmark for dried fruit, as freeze-drying is considered the gold standard for food preservation in the industry, removing 98–99% of all moisture [9].

## 2. Materials and Methods

### 2.1. Materials

Fresh pineapples (*Ananas comosus*) of a similar size, color, and ripeness were purchased from a local Fresh Thyme store. For comparison, fresh pineapple samples were used as a reference for untreated fruit. All chemicals for the nutritional analysis were used as received.

### 2.2. Passive Solar Drying and Freeze-Drying Systems’ Setup

**Scheme 1:**
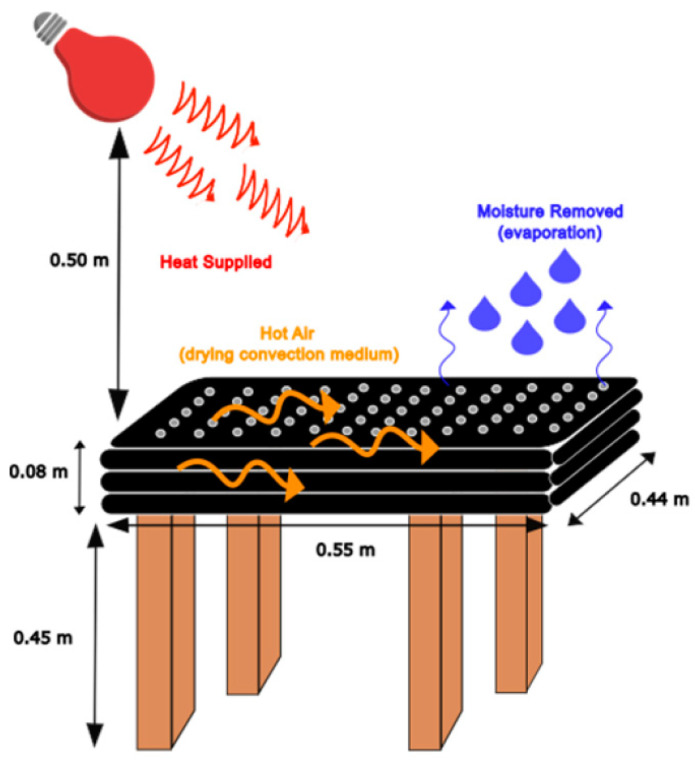
Setup of the passive solar drying system and the drying mechanism.

An Agricycle© (Milwaukee, WI, USA) solar drier (polyvinyl ether trays) and a HarvestRight™ freeze-dryer (Salt Lake City, UT, USA) were used for solar drying and freeze-drying, respectively. Fresh pineapples were peeled, cut, and sliced into thin wedge-shaped (~3 mm) pieces. The pieces (~250 g) were evenly placed on the top tray. Two 375-watt 125-volt R40 infrared heat lamps (catalog #03689-SU; Sunlite Manufacturing, Brooklyn, NY, USA) were used to simulate sunlight. Infrared light constitutes about 50% of solar energy and is responsible for thermal radiation and drying [5]. The system setup was housed in a psychrometric chamber, where the temperature (~30 °C) and relative humidity (~55%) are controlled and monitored (Scheme 1). Both temperature and humidity were constantly monitored. The parameters of the chamber were adjusted throughout the run, if necessary, to ensure they remained within their desired ranges. The samples were harvested after 16 h of drying. The 16 h time frame was determined based on a pilot study where the pineapple weight was monitored until there was no significant weight change. Similar to the solar-dried samples, the same amount of pineapple was placed on each of the trays and dried using a freeze-drier to obtain freeze-dried samples. During freeze-drying, the default settings were used. After a 15 min pre-cooling process, the samples were frozen at ~−23.3 °C, followed by vacuum freeze and drying (sublimation) at ≤500 mTorr and ~51.7 °C. After solar drying and freeze-drying, samples were collected, sealed in mylar bags, and stored at room temperature for further testing.

### 2.3. Water Content Analysis

Glass dishes were loaded with 5–10 pieces of pineapple wedges and placed into an oven preheated to 105 °C (Fisher Scientific™, Isotemp™, Waltham, MA, USA). The samples were monitored and weighed every 15 min for about 1 to 4 h depending on the sample, observing the color (ensuring the samples were not overheated) and weight change. Once the samples stopped losing mass, Equation (1) was used to determine the water content of the sample. This process was conducted for fresh, freeze-dried, and solar-dried samples, separately.

(1) $$Water\;Content=\frac{Initial\;Mass-Final\;Mass}{Initial\;Mass}\times 100\%,w.b.$$

### 2.4. Vitamin C, Total Phenolic Contents, Iron, and Dissolved Solids Analysis

The vitamin C testing was performed according to references [10,11]. Briefly, a standard curve was created by dissolving 10 mg ascorbic acid in 0.5% (*w*/*v*) oxalic acid solution and diluting with deionized (DI) water to a final concentration of 100 µg/mL. The solution was then diluted to 5 mL aliquots of 1, 4, 8, 12, and 16 µg/mL. A total of 0.5 mL of KMnO_4_ solution was added to each aliquot and the absorbance at 530 nm was measured using a UV–Vis Spectrophotometer (Genesys 150, Thermo Scientific; Waltham, MA, USA). For the pineapple samples, 10 g of each type of sample (fresh, freeze-dried, solar-dried) were ground and then mixed with 50 mL of 0.5% oxalic acid solution before being filtered through a Buchner funnel with Grade 1 Whatman paper. A minimum of 10 mL of solution was generated from each sample. A total of 1 mL of KMnO_4_ was added to 10 mL of each sample and mixed before the absorbance reading using a 1 cm × 1 cm cuvette.

The total phenolic contents of the samples were determined by the Folin–Ciocalteu method [12]. Briefly, pineapple extract was obtained by blending 5 g of pineapple in 50 mL of DI water. After the extract was centrifuged at 4000 rpm for 5 min at 4 °C, 20 μL of the supernatant was added to 1.58 mL DI water and 100 μL of the Folin–Ciocalteu reagent. A total of 300 μL of 20% sodium carbonate solution was then added to the mixture and vortexed for 10 min. The absorbance was measured at 765 nm using a UV–Vis Spectrophotometer after incubation in the dark for 2 h at room temperature. The standard curve was prepared using different concentrations of gallic acid and the results were expressed as mg gallic acid equivalent (GAE) per g sample.

The iron content testing methodology was adapted from [13]. To generate a ferric nitrate (Fe(NO_3_)_3_)/hydrochloric acid (HCl) standard curve, 0.00, 0.25, 0.50, 0.75, and 1.00 mM/L of Fe(NO_3_)_3_ in HCl solutions were prepared through a serial dilution of 0.001 M Fe(NO_3_)_3_ in HCl. To each 5 mL volume of Fe(NO_3_)_3_ in HCl solution, 2.5 mL of 0.10 M potassium thiocyanate (KSCN) solution was added. Absorbance at 458 nm was measured.

For the pineapple samples, 2.5 g of each sample was weighed and placed into crucibles. The crucibles were heated with a Bunsen burner for about 10–20 min until each sample turned to white/grayish ash, and then allowed to cool. Each cooled sample was mixed with 10 mL of 2.0 M HCl and stirred for 1 min, then 10 mL DI water was added and stirred for 1 min. A Buchner funnel with Whatman paper was used to filter out the solids. To the filtrate, 2.5 mL of 0.1 M KSCN was added and mixed well for the absorbance measurement.

An AiChose (Shenzhen, China) portable refractometer was used to measure the total dissolved solids in the samples. Five g of each pineapple sample were blended in 50 mL of phosphate-buffer solution (PBS). PBS was used as the blank. After blending, 1 mL of extract solution was placed on the refractometer screen and the reading was recorded.

### 2.5. Microbiological Analysis

Pineapple extract was obtained by blending 5 g of pineapple in 100 mL of PBS. After the extract was centrifuged at 4000 rpm for 5 min at 4 °C, the supernatant was used for analysis immediately. Three types of agar plates including standard plate count, MacConkey, and potato dextrose were inoculated with 500 µL of pineapple extract. The standard plate count agar (Oxoid Limited; Hampshire, UK) and MacConkey (Carolina Biological Supply Company; Burlington, NC, USA) plates were prepared according to the manufacturer’s instructions, while potato dextrose agar plates were purchased from Ward’s Science (Rochester, NY, USA). The microbial load was quantified for Gram-negative, total yeasts and molds, and total aerobic bacteria, respectively. *Escherichia coli* (#470176-528, Ward’s Science, Rochester, NY, USA) and *Saccharomyces cerevisiae* (Red Star Yeast Company, Milwaukee, WI, USA) were used as positive controls [5].

### 2.6. Fourier Transform Infrared (FTIR) Spectroscopy

Attenuated total reflection Fourier transform infrared (ATR-FTIR; Nicolet iS50, ThermoFisher Scientific; Waltham, MA, USA) spectroscopy was used to record the spectra of the samples against a background of air. The data were collected in the range 4000–650 cm^−1^ at room temperature [14,15]. OriginLab^®^ (Northampton, MA, USA) was then used to graph and analyze the spectra.

### 2.7. Color Analysis

Each type of pineapple was taken for colorimetry testing. A colorimeter (WR10QC Portable colorimeter; iWave Optoelectronics Technology Co., Ltd.; Shenzhen, China) was used to read the white/black, red/green, and yellow/blue values of each sample type per the manufacture manual. Various locations per piece of pineapple wedge and multiple pieces per sample were evaluated with a piece of white paper used as the blank. The color parameters were quantified by the color scale L* (whiteness), a* (redness (+) or greenness (−)), and b* (yellowness (+) or blueness (−)). The total color difference ΔE was used to indicate the overall color change and was calculated by using Equation (2):

(2) $$∆E=\sqrt{{\left(L^{*}-L_{0}^{*}\right)}^{2}+{\left(a^{*}-a_{0}^{*}\right)}^{2}+{\left(b^{*}-b_{0}^{*}\right)}^{2}}$$

where L_0_*, a_0_*, and b_0_* indicate the color parameters of the fresh samples. The average values of the color parameters were used for the calculation [16].

### 2.8. Microstructure Analysis

The surface microstructures of both freeze-dried and solar-dried samples were examined using scanning electron microscopy (SEM; JSM-6460LV, JEOL, Tokyo, Japan). The samples were placed on conductive carbon tape and then were coated with a thin layer (6–8 nm) of gold/palladium before imaging. The representative areas of the samples were imaged with an accelerating voltage of 5 kV [14,17].

### 2.9. Statistical Analysis 

All experiments were conducted in triplicates. Both ANOVA and *t*-tests were performed for water content and nutritional content testing and the results were considered statistically significant if *p* < 0.05. To normalize the collected data, Equation (3) was used. Normalization was performed for “fair” comparisons—considering more than 80% weight of fresh pineapple is water. Hence, normalization was only carried out to take dry mass (total mass—minus water content) into consideration.

(3) $$Normalized\;Measurment=\frac{Measurment}{Dry\;Mass\%}=\frac{Measurment}{100\%-Average\;Water\;Content},d.b.$$

## 3. Results and Discussion

### 3.1. Simulation of Sub-Saharan African Climate

**Figure 1:**
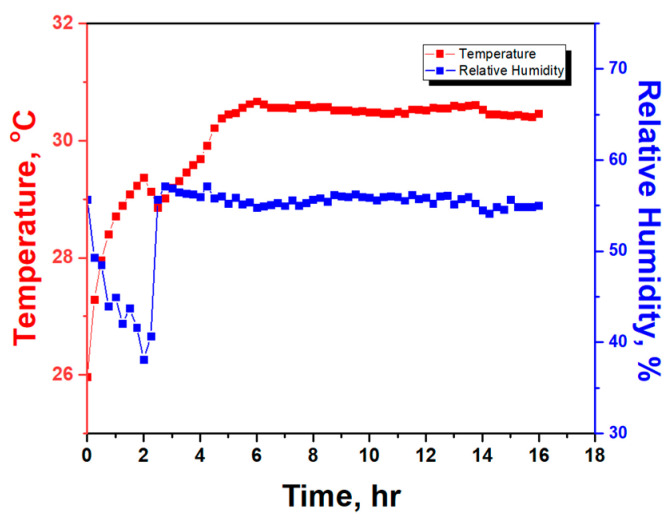
Temperature and humidity profile of the psychrometric chamber for the duration of a typical 16 h run time.

The Sub-Saharan African (SSA) climate is on average 26.7–32.2 °C and has an average relative humidity of 50–60% [18]. The psychrometric chamber was able to maintain the temperature and relative humidity of the SSA climate. As shown in Figure 1, after an initial 3 h acclimation period during start-up, the average temperature stabilized at ~30 °C and an average relative humidity of ~55%. Through the natural convection heat-transfer mechanism, the pineapple samples were uniformly dried, evaporating the water within the samples while raising the relative humidity within the chamber. Humidity levels fluctuated more at the early stage of the drying process, with most weight loss from the samples occurring during the first hours, as confirmed in the previous study [5]. The initial decrease in humidity (Figure 1) was most likely due to the large humidity difference between the chamber and the environment. Humidification was achieved using air-atomizing sprayers, which took time to reach the desired humidity. Moreover, the removal of water from the samples increased water vapor, which might have also affected humidity accumulation, especially in the early stages.

### 3.2. Water Content Post-Drying

**Figure 2:**
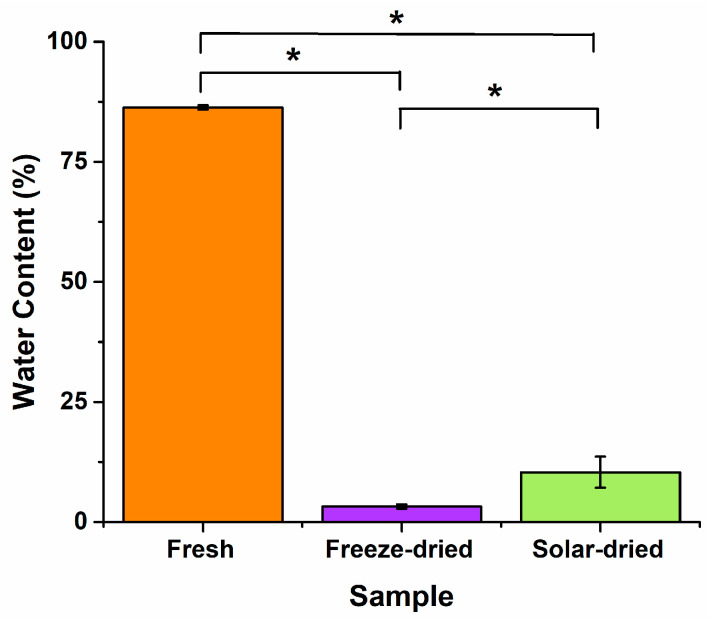
Water content of fresh, freeze-dried, and solar-dried pineapple samples. * indicates significant difference (*p* < 0.05).

Water content is an important factor to consider when assessing the safety and shelf stability of dried foods. Higher water contents have been shown to lead to increased microbial growth and hastened spoilage at ambient temperature, and within dried foods, fungus has been found to be the main driver of spoilage [19,20]. A lower water content correlates directly with improved shelf life and stability [20], the United Nations Economic Commission for Europe (UNECE) states that dried pineapples must contain less than 20% water in order for them to be qualified as safe dried food products [21]. Using an IsoTemp Oven, set at 105 °C, pineapple samples were fully dried to remove residue water without burning. The freeze-dried pineapple average water content was 3.21 ± 0.45%, while the solar-dried samples averaged around 10.4 ± 3.25%, meeting the standard of <20% (~half of the allowable amount). The fresh samples were used as a control to show the difference in the water content of a typical pineapple to a dried pineapple and showed a mean water content of 86.3 ± 0.44% (Figure 2).

### 3.3. Post-Drying Nutritional Content 

Various tests were conducted to verify that the pineapples were retaining their nutritional content during the drying process. Vitamin C, iron, and dissolved solids were measured. Vitamin C and iron were chosen because of their abundance within pineapple, as vitamin C is the most prevalent vitamin, and iron is the second most prevalent mineral within pineapples, according to the USDA [22]. Dissolved solids were used to measure the sugars present in the pineapples.

**Figure 3:**
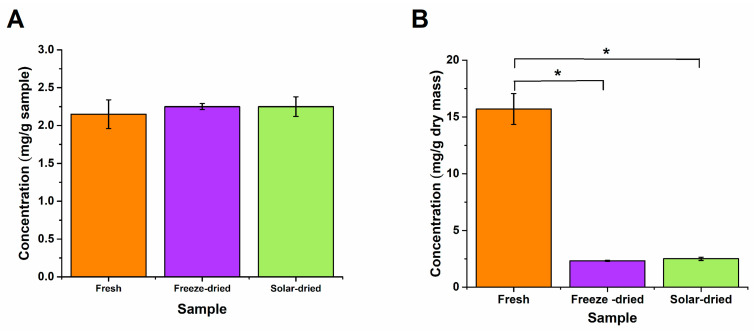
Vitamin C content of fresh, freeze-dried, and solar-dried samples. (**A**) raw data; (**B**) normalized for dry mass. * indicates significant difference (*p* < 0.05).

The raw data did not show any significant difference. However, the normalized data (per dry sample mass) showed significant differences (ANOVA: *p* = 1.15 × 10^−6^). The normalized vitamin C content within the fresh samples was the largest with 15.70 ± 1.36 mg/g sample; while the freeze-dried and solar-dried samples measured similarly at 2.33 ± 0.04 and 2.51 ± 0.14 mg/g sample respectively. Due to the nature of vitamin C, these results are as expected (Figure 3). Vitamin C, or ascorbic acid, has shown instability when faced with increased heat, moisture, oxygen, and pressure [23,24]. Ascorbic acid degrades by oxidation, hence the instability in the presence of moisture and oxygen, losing its physiological functions [23]. The degradation kinetics of ascorbic acid in the presence of heat have been shown to be linear, with degradation markedly increasing over time at temperatures ranging from 40 to 80 °C [23,25]. A slight increase was observed in the already high rates of degradation at temperatures of 80–90 °C, compared to a larger correlation shown with regard to surface area at temperatures of 60–80 °C. [23]. In addition, high pressure, similar to high heat, was shown to accelerate degradation [24]. Shelf temperature and drying time (affected by shelf temperature) can also significantly affect the vitamin C content during freeze-drying [26]. The high temperature (~75–80 °C) and high moisture (50–60% relative humidity) conditions within the tray presented by passive solar drying in a sub-Saharan like climate. These conditions combined with the large surface area to volume ratio of the thin pineapple slices would therefore create an ideal environment for Vitamin C degradation. Likewise, the high pressures observed in a freeze-drying cycle would likely also accelerate the degradation of Vitamin C.

**Figure 4:**
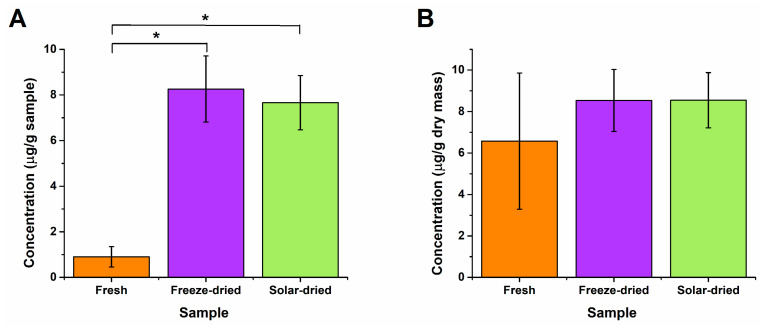
Iron content of fresh, freeze-dried, and solar-dried pineapple samples. (**A**) raw data; (**B**) normalized for dry mass. * indicates significant difference (*p* < 0.05).

**Figure 5:**
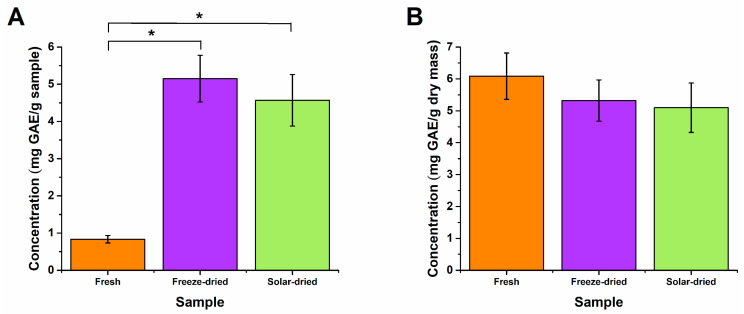
Total phenolic content of fresh, freeze-dried, and solar-dried pineapple samples. (**A**) raw data; (**B**) normalized for dry mass. * indicates significant difference (*p* < 0.05).

Regarding the iron content, the freeze-dried and solar-dried samples had much higher averages of 8.26 ± 1.44 and 7.66 ± 1.19 µg/g samples, respectively. The fresh sample measured the lowest at 0.90 ± 0.45 µg/g sample (Figure 4). However, upon normalization, no statistical difference among the three groups was determined. This is expected, as significant iron loss should not occur during drying. A previous study showed that improved solar drying could help retain minerals in pineapples compared to traditional solar drying methods [27]. A recent study showed that the iron content of the spray-dried strawberry powder was more than eight times higher compared to the fresh strawberries in wet weight [28]. As noticed in Figure 5, the fresh sample group has a larger error bar than the freeze-dried and solar-dried groups, which is due to the large water content, which complicated the measurement process.

Total phenolic content is one of the critical indicators of pineapple quality [6]. Although there was no significant statistical difference based on both the ANOVA and *t*-tests, the solar-dried samples showed the lowest average total phenolic content (5.10 ± 0.77 mg GAE/g dry mass) compared to 6.09 ± 0.73 mg GAE/g dry mass of fresh samples. High-temperature drying can lead to the inactivation of phenolic compounds and a reduction in phenolic substances by polyphenol oxidase [6,27].

**Figure 6:**
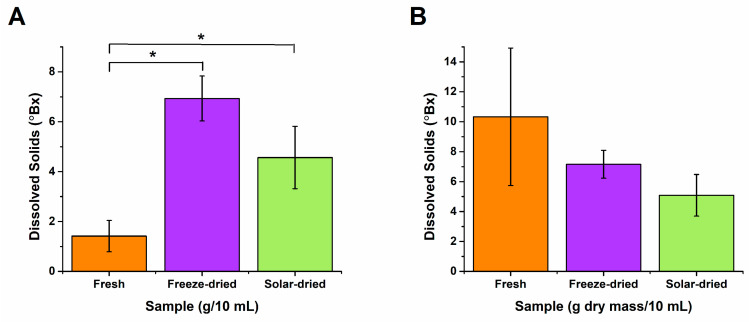
Dissolved solids content data for fresh, freeze-dried, and solar-dried pineapple samples. (**A**) raw data; (**B**) normalized for dry mass. * indicates significant difference (*p* < 0.05).

The quantity of dissolved solids is an important marker of the flavor and sweetness of the fruit. The dissolved solids include the total sugar content, the majority of which is glucose and fructose, in addition to some soluble proteins, amino acids, and other soluble organic materials [29]. The total dissolved solids of fruits and vegetables were highly influenced by the storage conditions [30,31]. Exposure to higher temperatures has been shown to lead to a loss in dissolved solids [32]. The total dissolved solids were determined using a portable refractometer. Upon normalization, the fresh sample showed the highest content at 10.3 ± 4.59 °Bx. The solar-dried sample had the lowest value at 5.09 ± 1.39 °Bx (Figure 6). However, no significant difference was observed through *t*-tests. The differences can be explained by the changes in the chemical and biochemical properties (such as carbohydrate conversion and biosynthesis) present in pineapple [30,31].

**Figure 7:**
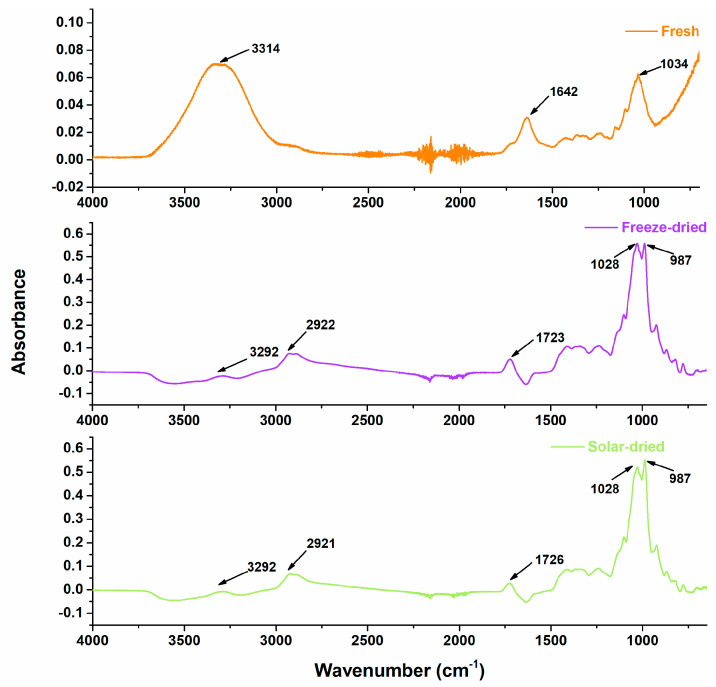
ATR-FTIR spectra of fresh, freeze-dried, and solar-dried pineapple samples.

ATR-FTIR was also performed to study the molecular modifications post drying [15]. As shown in Figure 7, the two major peaks at 3314 (O-H stretching) and 1642 cm^−1^ (O-H bending) are mostly attributed to water, while these peaks are much less pronounced for freeze-dried and solar-dried samples. The peaks within the region 1120–980 cm^−1^ are related to sugars and organic acids (coupled C-O (1028 cm^−1^) and C-C (987 cm^−1^) stretching). The peak around 1250 cm^−1^ indicates the symmetric stretching of aromatic nitro compounds and the C-O stretching of phenols. Moreover, the peaks around 2922 and 1723 cm^−1^ can be assigned to C-H and C=O stretching, respectively [15,33,34,35]. These peaks are dominant for both freeze-dried and solar-dried samples, indicating their relatively high percentage within the samples compared to the fresh ones. Overall, the spectra of freeze-dried and solar-dried samples are very close, indicating similar molecular compositions.

### 3.4. Post-Drying Microbiological Content 

**Figure 8:**
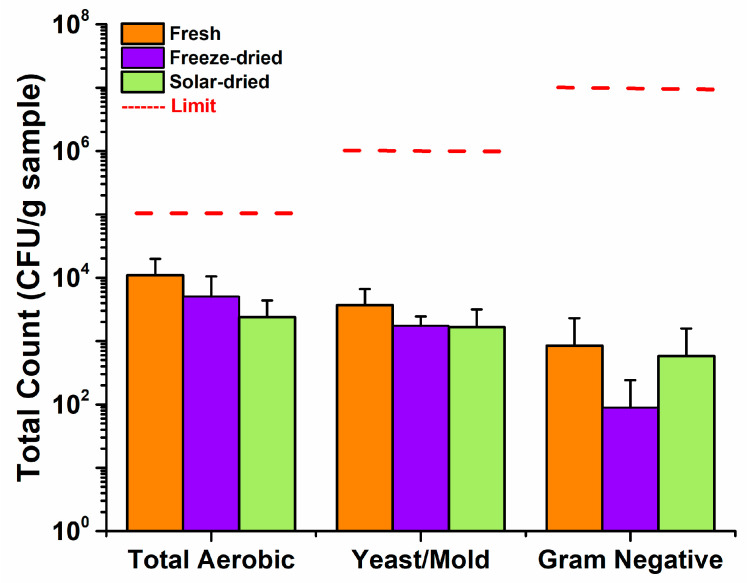
Microbial content for fresh, freeze-dried, and solar-dried pineapple samples. Dashed lines indicate the threshold for each category as permitted by several health agencies [42,43].

The drying process significantly reduces the water content and water activity, which largely prevents/postpones the growth of microorganisms. However, foods can be contaminated by microorganisms during the drying process [36]. Microbial safety is crucial for preserving minimally processed fruits [37]. For a comprehensive evaluation, microbiological content was determined for the following three types of microbes: aerobic bacteria, yeasts and molds, and Gram-negative bacteria. Plate count agar plates were used to determine total aerobic count, potato dextrose agar plates were used to determine total yeast/mold count, and MacConkey agar plates were used to determine the total Gram-negative count. Gram-negative bacteria were specifically determined, since they can cause serious diseases in humans and tend to be antibiotic-resistant [38]. For total aerobic bacteria, fresh samples exhibited the highest average CFU count of 1.54 × 10^4^, with the solar-dried samples exhibiting the lowest CFU count of 5.97 × 10^2^, and the freeze-dried samples containing 1.07 × 10^3^ CFU/g sample (Figure 8). In the yeast/mold category, fresh samples had the highest count with 5.22 × 10^3^ CFU/g sample. Solar-dried and freeze-dried samples had nearly the same counts of 4.15 × 10^2^ and 3.72 × 10^2^ CFU/g sample, respectively (Figure 8). Lastly, for the Gram-negative bacteria, fresh samples once again had the highest CFU count of 1.19 × 10^3^, with solar-dried samples having the second most count at 1.44 × 10^2^ CFU/g, and freeze-dried samples having the smallest count with 18.8 CFU/g sample (Figure 8). Overall, both drying processes did not contaminate the samples, as expected. For solar drying, the temperature of the tray could go up to ~75–80 °C which may contribute to microbial removal/inhibition. However, the drying process is not the same as sterilization. For example, little to no damage to microorganisms is observed during freeze-drying [36]. Instead, sources of produce and drying practices play vital roles in final product microbiological quality [39,40,41].

### 3.5. Post-Drying Color and Microstructure 

**Figure 9:**
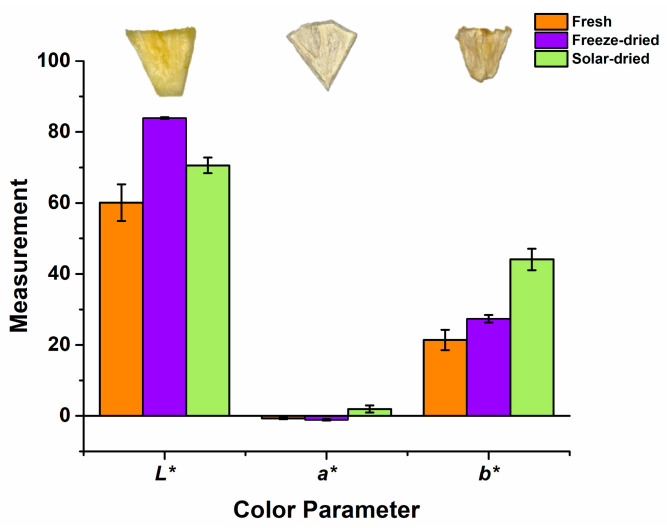
Color analysis data for fresh, freeze-dried, and solar-dried pineapple samples. Inserts are photos of fresh, freeze-dried, and solar-dried samples, respectively (left to right).

Color significantly affects the consumer’s choice and acceptance of food products [16,35]. The color evaluation showed that the freeze-dried sample exhibited the lightest color while the fresh samples were the darkest (Figure 9). The solar-dried samples were observed to have the strongest yellow with hints of red. The freeze-dried and fresh samples reflected more green than red and expressed less yellow than the solar-dried samples (Figure 9). The overall color difference (Δ*E*) was similar (24.6 vs. 25.1) for freeze-dried and solar-dried samples, as compared to the fresh samples. Color changes during drying can be attributed to the modifications in the exterior surface reflection, internal scattered light, and the facilitation of the loss of pigments. Prolonged heating and the presence of oxygen can lead to pigment oxidation and accompanying enzymatic browning [44].

**Figure 10:**
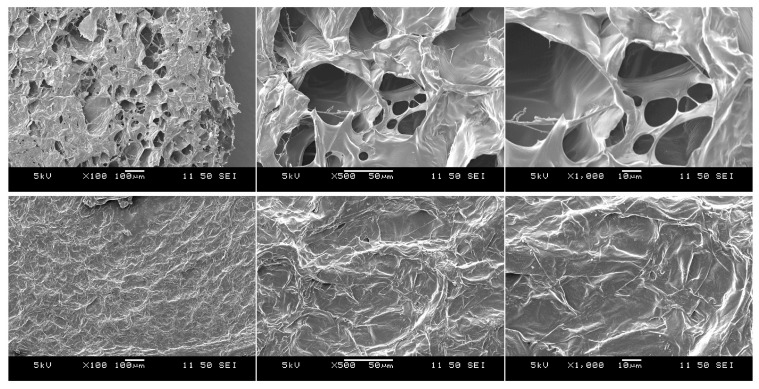
SEM images of freeze-dried (**top** panel) and solar-dried (**bottom** panel) pineapple samples. The magnification is 100, 500, and 1000 × from left to right, respectively.

Texture is one of the major factors for evaluating the overall quality of foods, which is an attribute derived from the molecular, microscopic and macroscopic structure of the food [3]. SEM images of the microstructure of the freeze-dried and solar-dried pineapples are shown in Figure 10. Fresh pineapple was not used for imaging due to its high water content. The two types of dried samples show very different structures. Freeze-dried pineapples showed a very porous structure. The formation of pores is a continuous process controlled by both the freezing and drying stages, and the porous characteristic is highly connected with the brittleness of freeze-dried products [3,45]. The solar-dried pineapple’s surface collapsed and the surface bulges were loosely arranged [17].

## 4. Conclusions

The effectiveness of passive solar drying has been demonstrated, compared to freeze-drying, as illustrated by the vitamin C, iron content, dissolved solids, colorimetry, and microbial testing results. These results indicated that the Agricycle^®^ passive solar dehydrator, during drying, retains major nutrients of pineapples such as vitamin C, total phenolic contents, and iron to levels comparable to the freeze-dried samples. However, the colors and microstructures were very different between freeze-dried and solar-dried samples. The water content of the solar-dried samples was less than 20% (~10%). The microbial loads were significantly below the limits for total aerobic, yeasts/molds, and Gram-negative microbes. Overall, as the results from the passive solar drying method meet the appropriate, acceptable limits (i.e., water content and microbial load) and the nutritional testing results are comparable to those from the freeze-dried control samples, the results validated the passive solar drying method using the Agricycle^®^ drier.

## Data Availability

The data that support the findings of this study are available from the corresponding author upon reasonable request.
